# Core Oligosaccharide of *Plesiomonas shigelloides* PCM 2231 (Serotype O17) Lipopolysaccharide—Structural and Serological Analysis

**DOI:** 10.3390/md11020440

**Published:** 2013-02-06

**Authors:** Anna Maciejewska, Jolanta Lukasiewicz, Marta Kaszowska, Aleksandra Man-Kupisinska, Wojciech Jachymek, Czeslaw Lugowski

**Affiliations:** 1 Department of Immunochemistry, Ludwik Hirszfeld Institute Immunology and Experimental Therapy, Polish Academy of Sciences, Rudolfa Weigla 12, 53-114 Wroclaw, Poland; E-Mails: czaja@iitd.pan.wroc.pl (J.L.); marta.kaszowska@iitd.pan.wroc.pl (M.K.); aleksandra.man@iitd.pan.wroc.pl (A.M.-K.); jachymek@iitd.pan.wroc.pl (W.J.); lugowski@iitd.pan.wroc.pl (C.L.); 2 Department of Biotechnology and Molecular Biology, University of Opole, Kardynala Kominka 6a, 45-032 Opole, Poland

**Keywords:** lipopolysaccharide, endotoxin, core oligosaccharide, *Plesiomonas shigelloides*

## Abstract

The herein presented complete structure of the core oligosaccharide of lipopolysaccharide (LPS) *P. shigelloides* Polish Collection of Microorganisms (PCM) 2231 (serotype O17) was investigated by ^1^H, ^13^C NMR spectroscopy, mass spectrometry, chemical analyses and serological methods. The core oligosaccharide is composed of an undecasaccharide, which represents the second core type identified for *P. shigelloides* serotype O17 LPS. This structure is similar to that of the core oligosaccharide of *P. shigelloides* strains 302-73 (serotype O1) and 7-63 (serotype O17) and differs from these only by one sugar residue. Serological screening of 55 strains of *P. shigelloides* with the use of serum against identified core oligosaccharide conjugated with bovine serum albumin (BSA) indicated the presence of similar structures in the LPS core region of 28 *O-*serotypes. This observation suggests that the core oligosaccharide structure present in strain PCM 2231 could be the most common type among *P. shigelloides* lipopolysaccharides.

## 1. Introduction

*Plesiomonas shigelloides* is the only species of the genus *Plesiomonas*. This rod-shaped Gram-negative enterobacterium [1] is known as the causative agent of water- and food-borne outbreaks of gastrointestinal infections. It was ranked third as a cause of travellers’ diarrhoea in Japan and China [2]. *P. shigelloides* is responsible for rare incidents of extra-intestinal infections in humans; most notably, meningitidis in neonates, bacteremia, sepsis and septic shock were reported for this bacterium. Sepsis and meningitidis caused by *P. shigelloides* are associated with serious cases and high fatality rate [2]. As a Gram-negative bacterium, *P. shigelloides* has a lipopolysaccharide (LPS, endotoxin) as a major component of the cell envelope that is also a main virulence factor. LPS is an amphiphilic molecule built of an *O*-specific polysaccharide (*O*-specific PS, *O*-antigen), core oligosaccharide (core OS) and lipid A. All these regions are significant for biological activities of LPS and are involved in host-bacterium interactions. Studies on the lipopolysaccharide structures of *P. shigelloides* are relatively new. To date only seven LPS structures out of 102 *O*-serotypes of *P. shigelloides* were investigated. As first structures, the *O*-specific polysaccharides of *P. shigelloides* strains 22074 and 12254 were determined in 1995 by Linnerborg *et al.* [3]. So far, only two complete LPS molecules isolated from *P. shigelloides* CNCTC 113/92 (serotype O54) and CNCTC 144/92 (serotype O74) [4,5,6,7,8] were elucidated. Additionally the structures of the core oligosaccharide substituted with the *O*-specific chains from strain 302-73 (serotype O1) [9,10,11] and the *O*-specific polysaccharides from strains CNCTC 110/92 (serotype O51) [12] and AM36565 [13] were identified. Although the number of publications concerning the structure of *P*. *shigelloides* lipopolysaccharide has increased since the year 2000, published data are still limited to seven strains only. All these studies showed a few characteristic features of *P. shigelloides* LPSs, that is, the lack of phosphate groups, the presence of uronic acid residue in the core oligosaccharide and the unusual hydrophobicity of the *O*-specific polysaccharides.

Recently, Kubler-Kielb *et al.* reported for the first time on the structure of the core oligosaccharide substituted with one repeating unit (RU) of the *O*-specific PS isolated from LPS of *P. shigelloides* strain 7-63 (serotype O17) [14]. It was known that its *O*-antigen structure is identical to that of *Shigella sonnei* phase I [15,16]—a causative agent of dysentery. It consists of a disaccharide biological repeating unit α-L-Alt*p*NAcA-(1→3)-β-D-Fuc*p*NAc4N [16]. Both species acquired virulence plasmid with gene cluster coding *O*-antigen of O17 serotype. Furthermore, strains of the serotype O17 of the genus *Plesiomonas* are the most frequently isolated from humans, mainly from cases of diarrhea [17].

We now present studies on the new type of core oligosaccharide identified for LPS of *P. shigelloides* strain Polish Collection of Microorganisms (PCM) 2231 classified as serovar O17:H11 [18]. Surprisingly, structures of the core OS of both strains differ in only one terminal sugar residue. For strain PCM 2231, terminal α-D-Glc*p*N was identified instead of terminal α-D-Gal*p*N in strain 7-63. The structure of the core oligosaccharide substituted with one repeating unit of the *O*-specific PS was investigated by ^1^H, ^13^C NMR spectroscopy, mass spectrometry, and chemical analyses. The serotype O17 was verified and a distribution of newly identified core oligosaccharide type was investigated with the use of rabbit polyclonal sera anti-core OS conjugate with bovine serum albumin (BSA).

## 2. Results and Discussion

### 2.1. Isolation of Lipopolysaccharide and Core Oligosaccharide

**Figure 1:**
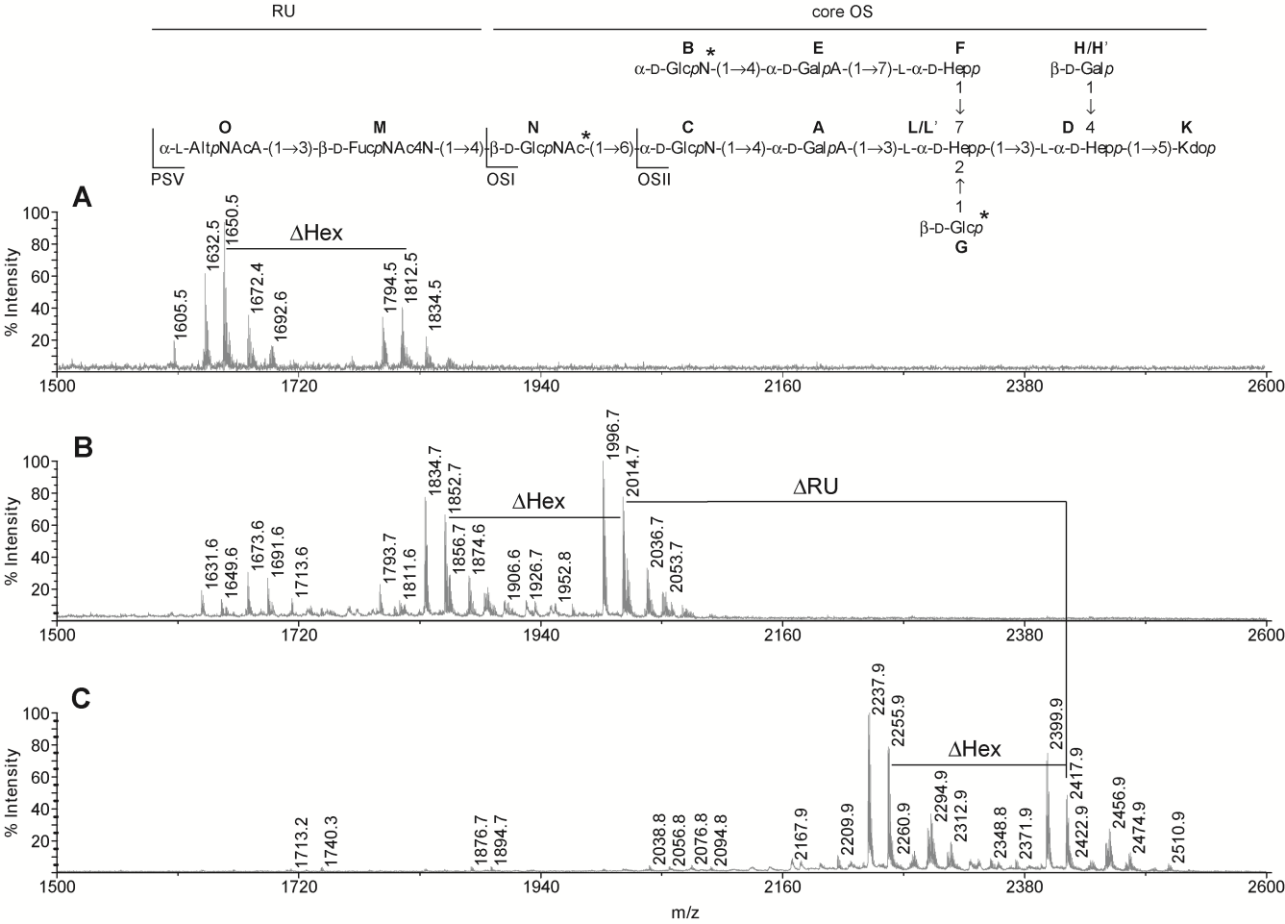
MALDI-TOF mass spectra of the core oligosaccharides OSII (**A**), OSI (**B**) and fraction PSV (**C**) isolated from LPS of *P. shigelloides* PCM 2231 (serotype O17). Complete structure of the core OS substituted with one RU of the *O*-specific PS was presented as the inset structure with marked OSII, OSI and PSV fractions. Heterogeneity related residues are marked with asterisk. Spectra were obtained in the negative reflectron mode with 2,4,6-trihydroxyacetophenone as a matrix. *m*/*z* values represent monoisotopic masses.

The lipopolysaccharide of *P. shigelloides* strain PCM 2231 (serotype O17) was isolated by phenol/water extraction and purified as previously reported [7]. Yield of LPS was 1.8% of the dry bacterial mass. The SDS-polyacrylamide gel electrophoresis (SDS-PAGE) analysis showed the smooth character of LPS, with a characteristic ladder-like pattern indicating different degree of polymerization of the *O*-antigen. The *O*-specific polysaccharides and different oligosaccharides were released by mild acid hydrolysis of the LPS (200 mg) and separated by gel filtration on Bio*-*Gel P-10. Five fractions of polysaccharide region: PSI (31.2 mg), PSII (3.7 mg), PSIII (3.1 mg), PSIV (3.8 mg), PSV (11.6 mg), and three fractions of oligosaccharide region: OSI (5.4 mg), OSII (2.0 mg) and OSIII (≤0.5 mg) were obtained (Figure 1, inset structure). Analysis of the fractions, with the use of MALDI-TOF MS, showed that PSII, PSIII, PSIV, and PSV consisted of the core OS substituted with four, three, two, and one repeating units of the *O*-specific PS, respectively. The PSI consisted of the core OS substituted with at least five RUs of the *O*-specific PS. Fraction OSIII was a mixture of low molecular weight oligosaccharides containing 3-deoxy-D-*manno*-oct-2-ulosonic acid (Kdo) released during mild acid hydrolysis of the LPS.

### 2.2. Chemical Analysis of the Oligosaccharides PSV and OSI

Initial NMR analysis of the OSI indicated the presence of uronic acid, Kdo, and two non-acetylated hexosamine residues. Thus all subsequent sugar and methylation analyses were carried out on *N*-acetylated and carboxyl-reduced oligosaccharide to detect these residues. Compositional analysis of the *N*-acetylated core oligosaccharide (OSI) together with determination of the absolute configuration revealed the presence of L,D-Hep, D-Glc, D-Gal, D-GalA, and D-GlcN. Methylation analysis performed on the carboxyl-reduced and *N*-acetylated OSI indicated the presence of 2,3,7-trisubstituted L,D-Hep*p*, 3,7-disubstituted L,D-Hep*p*, 3,4-disubstituted L,D-Hep*p*, 7-substituted L,D-Hep*p*, terminal D-Glc*p*N, 6-substituted D-Glc*p*N, terminal D-Glc*p*, terminal D-Gal*p*, 4-substituted D-Gal*p*A, and 5-substituted Kdo. The PSV fraction consisted of all residues identified for OSI, and additionally 4-substituted D-Glc*p*N and terminal Alt*p*NAcA were detected.

### 2.3. MALDI-TOF MS Analyses of the PSV, OSI and OSII

The MALDI-TOF mass spectrum of the OSI showed two main clusters of two pairs of ions at *m*/*z* 2014.7 [M − H]^−^ and 1996.7 [M − H_2_O − H]^−^ and *m*/*z* 1852.7 [M − Hex − H]^−^ and 1834.7 [M − Hex − H_2_O − H]^−^ (Figure 1B and inset structure). The ion at *m*/*z* 2014.7 corresponded to the complete core OS built of three Hep, Glc, Gal, two GalA, two GlcN, GlcNAc, and Kdo, which gave together a calculated monoisotopic mass of 2015.7 Da. The ion at *m*/*z* 1852.7 [M − Hex − H]^−^ corresponded to a decasaccharide devoid of one hexose residue in comparison with the complete core oligosaccharide. Less abundant ions at *m*/*z* 1811.6, *m*/*z* 1691.6, and *m*/*z* 1649.6 represent the complete core OS devoid of *N*-acetylohexosamine [M − HexNAc − H]^−^, both hexose and hexosamine [M − Hex − HexN − H]^−^, and both hexose and *N*-acetylohexosamine residues [M − Hex − HexNAc − H]^−^, respectively.

The mass spectrum of the oligosaccharide OSII was obtained for fraction analysed by NMR prior to mass spectrometry (Figure 1A and inset structure). *m*/*z* values of observed ions were ~1 Da higher than calculated masses of ions as a result of incomplete exchange of deuterium to hydrogen. Therefore major ions at *m*/*z* 1812.5 and 1650.5 corresponded to the core oligosaccharide devoid of one *N*-acetylohexosamine residue [M − HexNAc − H]^−^ and both *N*-acetylohexosamine and hexose residues [M − Hex − HexNAc − H]^−^. Less abundant ions at *m*/*z* 1834.5 [M − HexNAc + Na − H]^−^, 1672.4 [M − Hex − HexNAc + Na − H]^−^, 1794.5 [M − HexNAc − H_2_O − H]^−^ and 1632.5 [M − Hex − HexNAc − H_2_O − H]^−^ represent the monosodiated or dehydrated molecules, respectively. The mass spectrum of the isolated PSV fraction (Figure 1C and inset structure) showed main ions at *m*/*z* 2417.9 [M − H]^−^, *m*/*z* 2399.9 [M − H_2_O − H]^−^ supported a tridecasaccharide structure built of one disaccharide repeating unit of the *O*-specific PS (mass difference of 403.2 Da) linked to the complete core OS. Ions at *m*/*z* 2255.9 and 2237.9 corresponded to the structure devoid of one hexose, resembling previously reported heterogeneity of the OSI and OSII. Finally, the presence of glycine was suggested by ions at *m*/*z* 2294.9, 2312.9, 2456.9, and 2474.9 separated by 57 Da with reference to corresponding ions at *m*/*z* 2237.9, 2255.9, 2399.9, and 2417.9.

### 2.4. NMR Analysis of the PSV

**Figure 2:**
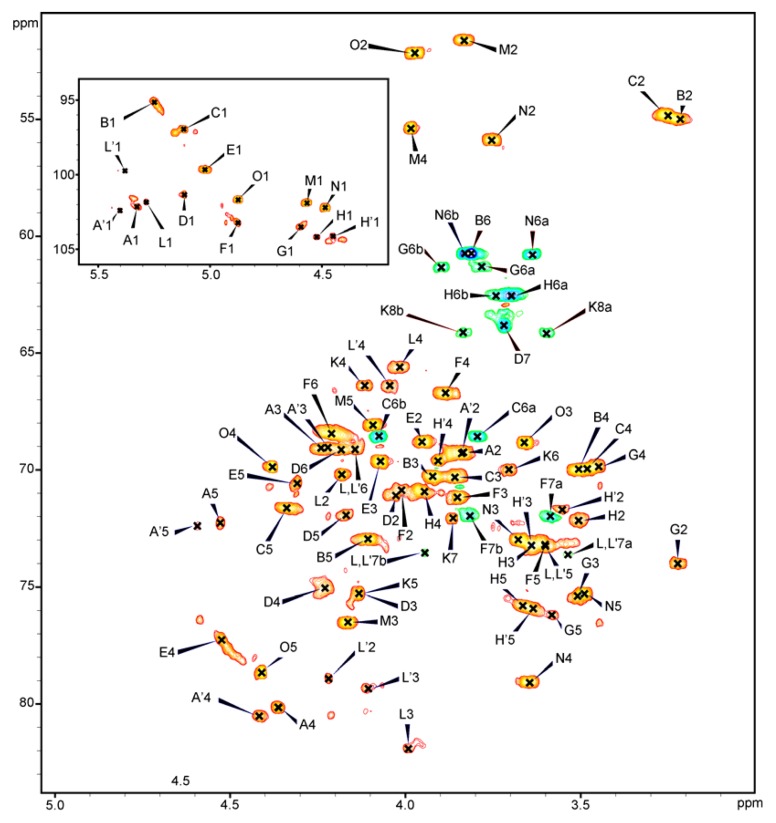
.600 MHz HSQC-DEPT spectrum of the core OS substituted with one RU of the *O*-specific PS of *P. shigelloides* PCM 2231 (serotype O17) (fraction PSV). The inset shows the anomeric region of the spectrum. The uppercase letters refer to designations of carbohydrate residues. The spectra were obtained for ^2^H_2_O suspensions at 27 °C.

The fraction PSV consisted of the core oligosaccharide substituted with one repeating unit of the *O*-specific chain was analysed by 1D and 2D ^1^H, ^13^C-NMR spectroscopy. All the spin systems (Table 1) were assigned by COSY, TOCSY with different mixing times, HSQC-DEPT (Figure 2), HSQC-TOCSY, NOESY and HMBC spectra. Experimental chemical shift values were compared with previously published NMR data for respective monosaccharides and oligosaccharides [8,14,19].

**Table 1:** ^1^H and ^13^C NMR chemical shifts of the core oligosaccharide substituted with one RU of the *O*-specific PS of *P. shigelloides* PCM 2231 LPS (serotyp O17) (fraction PSV). Spectra were recorded for ^2^H_2_O solution at 27 °C. Acetone (δ_H_ 2.225, δ_C_ 31.05 ppm) was used as internal reference.

Residue		Chemical shifts (ppm)							
		H-1	H-2	H-3a,b	H-4	H-5	H-6a,b	H-7a,b	H-8a,b
		C-1	C-2	C-3	C-4	C-5	C-6	C-7	C-8, CH_3_CO
A	→4)-α- D -Gal *p* A-(1→	5.33	3.83	4.25	4.36	4.53			
		102.1	69.2	69.0	80.1	72.3	175.6		
A′	→4)-α- D -Gal *p* A-(1→	5.40	3.84	4.22	4.41	4.60			
		102.4	69.3	69.0	80.6	72.5	175.6		
B	α- D -Glc *p* N-(1→	5.24	3.22	3.91	3.50	4.11	3.81 ^a^		
		95.1	55.0	70.3	70.0	73.0	60.7		
C	→6)-α-D-Glc*p*N-(1→	5.12	3.26	3.86	3.47	4.33	3.79, 4.08		
		97.0	54.9	70.3	70.0	71.6	68.6		
D	→3,4)-L-α-D-Hep*p*-(1→	5.11	4.04	4.13	4.23	4.17	4.17	3.72 ^b^	
		101.3	71.1	75.3	75.1	72.0	69.2	63.8	
E	→4)-α-D-Gal*p*A-(1→	5.02	3.95	4.07	4.52	4.31			
		99.7	68.8	69.7	77.3	70.6	176.5		
F	→7)-L-α-D-Hep*p*-(1→	4.88	4.00	3.84	3.89	3.60	4.21	3.58, 3.82	
		103.2	70.9	71.2	66.7	73.2	68.4	72.0	
G	β-D-Glc*p*-(1→	4.59	3.22	3.51	3.45	3.59	3.78, 3.90		
		103.5	74.0	75.4	69.9	76.2	61.3		
H	β-d-Gal*p*-(1→	4.51	3.50	3.62	3.95	3.66	3.69-3.74 ^b^		
		104.2	72.2	73.1	71.0	75.8	62.6		
H′	β-d-Gal*p*-(1→	4.45	3.55	3.64	3.91	3.64	3.69-3.74 ^b^		
		104.1	71.7	73.2	69.6	76.0	62.6		
K	→5)-Kdo			1.86, 2.22	4.12	4.13	3.70	3.86	3.60, 3.84
		nd	nd	34.4	66.4	75.3	70.0	72.1	64.1
L	→3,7)-L-α-D-Hep*p*-(1→	5.28	4.18	3.99	4.01	3.60	4.14	3.55, 3.95	
		101.8	70.2	81.9	65.6	73.2	69.1	73.5	
L′	→2,3,7)-L-α-D-Hep*p*-(1→	5.38	4.22	4.10	4.04	3.60	4.14	3.56, 3.94	
		99.7	78.9	79.3	66.4	73.2	69.1	73.6	
M	→3)-β-d-Fuc*p*NAc4N-(1→	4.57	3.83	4.16	3.98	4.09	1.32		2.07
		101.9	51.6	76.5	55.4	68.1	16.3		23.0, 174.7
N	→4)-β-D-Glc*p*NAc-(1→	4.48	3.75	3.68	3.65	3.49	3.63, 3.82		2.06
		102.2	55.9	73.0	79.1	75.3	60.8		23.0, 175.4
O	α-l-Alt*p*NAcA	4.88	3.97	3.66	4.38	4.41			2.00
		101.7	52.2	68.8	69.9	78.7	175.4		23.0, 175.3

^a, b ^Not resolved; nd: Not determined.

The HSQC-DEPT spectrum obtained for PSV fraction contained signals for twelve major anomeric protons and carbons, and a Kdo spin system (Figure 2, Table 1).

Residue **A** with the H-1/C-1 signals at δ 5.33/102.1 ppm, *J*_C-1,H-1_ ~176 Hz as well as residue **E** with the H-1/C-1 signals at δ 5.02/99.7 ppm, *J*_C-1,H-1_ ~176 Hz, were assigned as the 4-substituted α-D-Gal*p*A residues based on the characteristic five proton spin systems, the relatively high chemical shifts of the H-5, H-4, and H-3, the high ^13^C chemical shift of the C-4 (residue **A**: δ 80.1, residue **E**: δ 77.3) and C-6 (residue **A**: δ 175.6, residue **E**: δ 176.5) signals, large vicinal couplings between H-2, H-3 and the small vicinal couplings between H-3, H-4, and H-5 protons. Residue **A**′ was also identified due to heterogeneity (see text below) (Table 1).

Residue **B** with the H-1/C-1 signals at δ 5.24/95.1 ppm, *J*_C-1,H-1_ ~176 Hz, was assigned as the terminal α-D-Glc*p*N based on the chemical shift of the C-2 (δ 55.0), coupling constants between all ring protons.

Residue **C** with the H-1/C-1 signals at δ 5.12/97.0 ppm, *J*_C-1,H-1_ ~175 Hz, was recognized as the 6-substituted α-D-Glc*p*N based on the chemical shift of the C-2 (δ 54.9) and coupling constants between all ring protons and the characteristic downfield shift of the C-6 signal (δ 68.6).

Residue **D** with the H-1/C-1 signals at δ 5.11/101.3 ppm, *J*_C-1,H-1_ ~173 Hz, was recognized as a 3,4-disubstituted L-*glycero*-α-D-*manno*-Hep*p* based on coupling constants among H-1, H-2, and H-3 and the relative high chemical shift of the C-3 (δ 75.3) and C-4 (δ 75.1) signals.

Residue **F** with the H-1/C-1 signals at δ 4.88/103.2 ppm, *J*_C-1,H-1_ ~176 Hz, was recognized as the 7-substituted L-*glycero*-α-D-*manno*-Hep*p* from the ^1^H and ^13^C chemical shifts, the small vicinal couplings between H-1, H-2 and H-3 and similar chemical shifts as those for the monosaccharide L-α-D-Hep*p* and the relative high chemical shift of the C-7 (δ 72.0).

Residue **G** with the H-1/C-1 signals at δ 4.59/103.5 ppm, *J*_C-1,H-1_ ~163 Hz, was assigned as the terminal β-D-Glc*p* based on coupling constants between all protons in the sugar ring.

Residue **H** with the H-1/C-1 signals at δ 4.51/104.2 ppm, *J*_C-1,H-1_ ~163 Hz, was recognized as the terminal β-D-Gal*p* due to the large coupling between H-1, H-2, and H-3 and the small vicinal couplings among H-3, H-4, and H-5. Residue **H**′ was also identified due to heterogeneity of the structure (see text below) (Table 1).

Residue **K** was identified as a 5-substituted Kdo based on the characteristic deoxy proton signals of H-3a (δ 1.86 ppm), H-3b (δ 2.22 ppm) and a high chemical shift of the C-5 signal (δ 75.3 ppm).

Residue **L**′ with the H-1/C-1 signals at δ 5.38/99.7 ppm, *J*_C-1,H-1_ ~176 Hz, was recognized as the 2,3,7-trisubstituted L-*glycero*-α-D-*manno*-Hep*p* based on ^1^H and ^13^C chemical shifts, the small vicinal couplings between H-1, H-2, and H-3, and the relatively high chemical shifts of the C-2 (δ 78.9), C-3 (δ 79.3), and C-7 (δ 73.6) signals. Residue **L** was also identified due to heterogeneity (see text below) (Table 1).

Residue **M** with the H-1/C-1 signals at δ 4.57/101.9 ppm, *J*_C-1,H-1_ ~168 Hz, was assigned as the 3-substituted β-D-Fuc*p*NAc4N based on the characteristic signal of the exocyclic CH_3_ group (δ_H_ 1.32, δ_C_ 16.3), the chemical shift of the C-2 (δ 51.6) and C-4 (δ 55.4) signals, the downfield shift of the C-3 signal (δ 76.5), and small vicinal couplings between H-3, H-4 and H-5. Residue **M** is a constituent of the *O*-specific PS disaccharide RU.

Residue **N** with the H-1/C-1 signals at δ 4.48/102.2 ppm, *J*_C-1,H-1_ ~163 Hz, was recognized as the 4-substituted β-D-Glc*p*NAc based on the chemical shift of the C-2 signal (δ 55.9), the relative high chemical shift of the C-4 signal (δ 79.1) and coupling constants between all ring protons.

Residue **O** with the H-1/C-1 signals at δ 4.88/101.7 ppm, *J*_C-1,H-1_ ~167 Hz, was assigned as the terminal α-L-Alt*p*NAcA based on the chemical shift of the C-2 signal (δ 52.2), the high ^13^C chemical shift of the C-6 (δ 175.4) signal. Residue **O** is a constituent of the *O*-specific PS disaccharide RU.

Three additional anomeric signals and spin systems were present in all NMR spectra. Residue **L** with the H-1/C-1 signals at δ 5.28/101.8 ppm, *J*_C-1,H-1_ ~176 Hz, was recognized as the 3,7-disubstituted L-*glycero*-α-D-*manno*-Hep*p* from the ^1^H and ^13^C chemical shifts, the small vicinal couplings between H-1, H-2, and H-3, and the relatively high chemical shifts of the C-3 (δ 81.9) and C-7 (δ 73.5) signals. Residue **L** represented disubstituted variant of residue **L**′ (2,3,7-trisubstituted L-*glycero*-α-D-*manno*-Hep*p*) caused by the lack of the β-D-Glc*p* residue at *O*-2 of **L**′ residue. Residues **A**′ (H-1/C-1 signals at δ 5.40/102.4 ppm) and **H**′ (H-1/C-1 signals at δ 4.45/104.1 ppm) were recognized as variants of residue **A** (4-substituted α-D-Gal*p*A) and **H** (terminal β-D-Gal*p*), respectively, due to the absence of the β-D-Glc*p* residue.

The inter-residue connectivities between the adjacent monosaccharides were observed by NOESY and HMBC experiments (Table 2). Each disaccharide element in the PSV was identified, providing the sequence of monosaccharides. For PSV inter-residue Nuclear Overhauser Effects (NOEs) were found between H-1 of **O** and H-3 of **M**, H-1 of **M** and H-4 of **N**, H-1 of **N** and H-6a,b of **C**, H-1 of **C** and H-4 of **A**, H-1 of **A** and H-3 of **L**, H-1 of **L** and H-3 of **D**, H-1 of **D** and H-5 of **K**, H-1 of **B** and H-4 of **E**, H-1 of **E** and H-7a,b of **F**, H-1 of **F** and H-7a,b of **L/L**′, H-1 of **G** and H-2 of **L**′ and H-1 of **H**/**H**′ and H-4 of **D**.

**Table 2:** Selected ^3^*J*_H,C_-connectivities from the anomeric atoms of the core OS substituted with one RU of the *O*-specific PS of *P. shigelloides* PCM 2231 LPS (serotype O17).

Residue		Atom H-1/C-1 (ppm)	Connectivities to		Inter-Residue atom/residue
			δ_H_	δ_C_	
A	→4)-α-D-Gal*p*A-(1→	5.33/102.1	3.99	nd	H-3 of **L**
B	α-D-Glc*p*N-(1→	5.24/95.1	4.52	nd	H-4 of **E**
C	→6)-α-D-Glc*p*N-(1→	5.12/97.0	4.36	80.1	H-4, C-4 of **A**
D	→3,4)-L-α-D-Hep*p*-(1→	5.11/101.3	4.13	75.3	H-5, C-5 of **K**
E	→4)-α-D-Gal*p*A-(1→	5.02/99.7	3.58	71.9	H-7a, C-7 of **F**
F	→7)-L-α-D-Hep*p*-(1→	4.88/103.2	3.95	73.5	H-7b, C-7 of **L**,**L′**
G	β-D-Glc*p*-(1→	4.59/103.5	nd	78.9	C-2 of **L**′
H	β-D-Gal*p*-(1→	4.51/104.2	4.23	75.1	H-4, C-4 of **D**
L	→3,7)-L-α-D-Hep*p*-(1→	5.28/101.2	4.13	75.3	H-3, C-3 of **D**
M	→3)-β-D-Fuc*p*NAc4N-(1→	4.57/101.9	3.65	79.1	H-4, C-4 of **N**
N	→4)-β-D-Glc*p*NAc-(1→	4.48/102.2	3.79, 4.08	68.6	H-6a, H-6b, C-6 of **C**
O	α-l-Alt*p*NAcA	4.88/101.7	4.16	76.5	H-3, C-3 of **M**

nd: Not determined.

In the fraction PSV, glycine was identified by the presence of an additional carbonyl resonance at δ_C_ 168.7 ppm in the HMBC spectrum and a negative CH_2_ crosspeak (δ_H_ 3.98 ppm, δ_C_ 40.9 ppm) in the HSQC-DEPT spectrum. The presence of glycine in the PSV component was further supported by amino acid analysis and mass spectrometry; however connectivity between the glycine and the oligosaccharide was not determined.

These studies demonstrate the structure of the complete core oligosaccharide and linkage between the core region and *O*-specific PS of lipopolysaccharide *P. shigelloides* PCM 2231 (serotype O17). The structure of the complete core region of *P. shigelloides* PCM 2231 is similar to that of the core OSs of *P. shigelloides* strains 7-63 (serotype O17) [14] and 302-73 (serotype O1) [10]. The core OS of strain 7-63 (serotype O17) differs only by a single terminal residue of branched chain, that is, terminal α-D-Gal*p*N was present instead of the terminal α-D-Glc*p*N (residue **B**) in strain PCM 2231 (serotype O17). The core OS of *P. shigelloides* 302-73 (serotype O1) contains Kdo as a residue linking the *O*-specific polysaccharide with the outer core region instead of β-D-Glc*p*NAc (residue **N**) present in strain PCM 2231 (serotype O17).

### 2.5. Serological Studies

**Figure 3:**
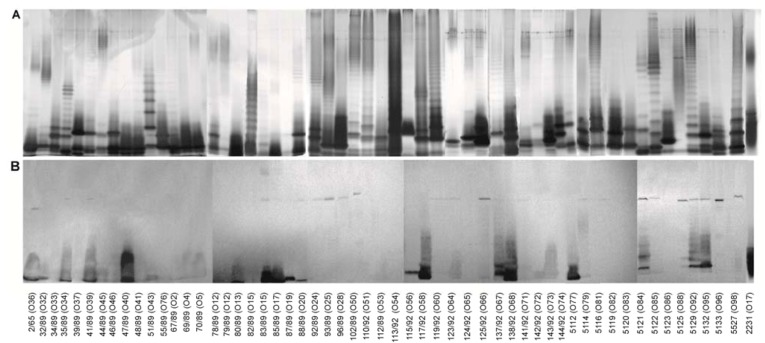
LPSs were analysed by SDS-PAGE (3 μg/lane), using a 15% separating gel, and visualized by the silver staining method (**A**). Reactivities of serum (200-fold diluted) against the OSI-BSA conjugate with *P. shigelloides* LPSs in immunoblotting (**B**).

The core oligosaccharide (OSI) was conjugated with BSA (OSI-BSA) with the use of high temperature conjugation described by Boratynski *et al.* [20]. The polyclonal antisera obtained by immunization of rabbits with the conjugate were used to scan LPSs isolated from 55 strains of *P. shigelloides* for the presence of epitopes similar to those found in LPS of *P. shigelloides* PCM 2231 (serotype O17). The obtained conjugate was a good immunogen inducing high level of specific anti-core OS antibodies as was shown in a solid-phase ELISA with homologous LPS as an antigen (data not shown). LPSs were isolated by hot phenol/water extraction. Most of them were recovered from both the water and the phenol phase. LPSs were separated by SDS-PAGE (Figure 3A) and stained using the method of Tsai [21]. The reactivities of anti-conjugate sera with LPSs isolated from various *P*.* shigelloides* strains were examined with the use of immunoblotting (Figure 3B). Results for LPSs which were recovered from the phenol phases were identical in comparison with the water phase derived LPSs (data not shown). To simplify presented data, results for the water phase derived LPSs were shown with the exception of LPSs CNCTC 39/89, 102/89, 5112, recovered from the phenol phase.

The antibodies against the OSI-BSA conjugate reacted mainly with fast migrating LPS fractions, representing LPS with unsubstituted core oligosaccharide. However reactions with core OS epitopes present in smooth lipopolysaccharides were also observed. Serological screening of different strains of *P. shigelloides* indicates that similar epitopes might also be present in the core OS of the 28 out of 55 strains. Intensities of cross-reactions observed for different LPSs were diversified. Strong cross-reactions were observed with the fast migrating LPS fractions of strains CNCTC 2/65, 35/89, 41/89, 47/89, 80/89, 83/89, 85/89, 87/89, 88/89, 115/92, 117/92, 137/92, 138/92, 5112, 5121, 5129, and 5132 (Figure 3). Weak reactions were observed for LPSs *P. shigelloides* CNCTC 32/89, 51/89, 69/89, 70/89, 78/89, 123/92, 125/92, 142/92, 143/92, 5114, and 5527.

## 3. Experimental Section

### 3.1. Bacteria

*P. shigelloides* strain PCM 2231 (serovar O17:H11) was obtained from the Polish Collection of Microorganisms (PCM) of the Institute of Immunology and Experimental Therapy, Wroclaw, Poland and 55 available different *P. shigelloides* strains CNCTC 2/65 (O36), 32/89 (O32), 34/89 (O33), 35/89 (O34), 39/89 (O37), 41/89 (O39), 44/89 (O45), 46/89 (O46), 47/89 (O40), 48/89 (O41), 51/89 (O43), 55/89 (O76), 67/89 (O2), 69/89 (O4), 70/89 (O5), 78/89 (O12), 79/89 (O12), 80/89 (O13), 82/89 (O15), 83/89 (O15), 85/89 (O17), 87/89 (O19), 88/89 (O20), 92/89 (O24), 93/89 (O25), 96/89 (O28), 102/89 (O50), 110/92 (O51), 112/92 (O53), 113/92 (O54), 115/92 (O56), 117/92 (O58), 119/92 (O60), 123/92 (O64), 124/92 (O65), 125/92 (O66), 137/92 (O67), 138/92 (O68), 141/92 (O71), 142/92 (O72), 143/92 (O73), 144/92 (O74), 5112 (O77), 5114 (O79), 5116 (O81), 5119 (O82), 5120 (O83), 5121 (O84), 5122 (O85), 5123 (O86), 5125 (O88), 5129 (O92), 5132 (O95), 5133 (O96), 5527 (O98) were obtained from the collection of the Institute of Hygiene and Epidemiology, Prague, Czech Republic. Bacteria were grown and harvested as described previously [22].

### 3.2. Lipopolysaccharide and Core Oligosaccharide

The LPS was extracted from bacterial cells by the hot phenol/water method [23] and purified as previously reported [7]. The yield of LPS was ~1.8% of the dry bacterial mass. LPS (200 mg) was degraded by treatment with 1.5% acetic acid containing 2% SDS at 100 °C for 15 min. The reaction mixture was freeze-dried, the SDS removed by extraction with 96% ethanol, and the residue suspended in water and centrifuged. The poly- and oligosaccharides were separated by gel permeation chromatography, performed on Bio-Gel 10 column, equilibrated with 0.05 M pyridine/acetic acid buffer of pH 5.6. Eluates were monitored with a Knauer differential refractometer and all fractions were freeze-dried and checked by MALDI-TOF mass spectrometry. 

### 3.3. Analytical Methods

Monosaccharides were analysed as their alditol acetates by GC-MS [24]. Absolute configurations of the monosaccharides were determined as described by Gerwig *et al.* [25,26] using (−)-2-butanol for the formation of 2-butyl glycosides. The trimethylsilylated butyl glycosides produced from authentic samples were used as standards. Carboxyl reduction of the core oligosaccharide fractions was carried out according to the method of Taylor *et al.* [27] as previously described [22]. Methylation analyses were performed on both native and carboxyl reduced oligosaccharides according to the method of Hakomori [28]. Alditol acetates and partially methylated alditol acetates were analysed with a Hewlett-Packard 5972 system using the HP-1 fused-silica capillary column (0.2 mm × 12.5 m), He as carrier gas, flow rate 1 mL·min^−1^ and a temperature program 150 → 270 °C at 8 °C·min^−1^.

### 3.4. Mass Spectrometry

MALDI-TOF MS spectra (reflectron negative-ion mode) were obtained on a MDS SCIEX 4800 MALDI TOF/TOF and Bruker Autoflex III Tof/Tof instruments. Samples were dissolved in water (0.5 mg/mL) and desodiated with Dowex 50 W × 8 (H^+^). 2,4,6-Trihydroxyacetophenone (25 mg/mL in acetonitrile:water, 1:1, v/v) was used as a matrix.

### 3.5. NMR Spectroscopy

All NMR spectra were obtained on Bruker 600 MHz spectrometer (Laboratory of Structural Analyses, Wroclaw University of Technology, Wroclaw, Poland). The fraction PSV containing the core oligosaccharides substituted with one repeating unit of the *O*-specific chain was first repeatedly exchanged with ^2^H_2_O (99.9%) with intermediate lyophilisation. NMR spectra were obtained for ^2^H_2_O solutions at 27 °C using acetone (δ_H_ 2.225 ppm, δ_C_ 31.05 ppm) as internal reference. The signals were assigned by one- and two-dimensional experiments (COSY, TOCSY, NOESY, HMBC, HSQC-DEPT and HSQC-TOCSY). The *J*_C-1,H-1_ values were obtained from non-decoupled HSQC-DEPT experiment. In the TOCSY experiments the mixing times were 60, 90 and 120 ms. The delay time in the HMBC was 60 ms and the mixing time in the NOESY experiment was 60 ms.

All spectra were acquired and processed using standard Bruker software. The processed 2D spectra were assigned using the SPARKY program [29].

### 3.6. Preparation of Oligosaccharide Conjugate with BSA

The core oligosaccharide (OSI) was oxidised and purified as describe previously [30]. Briefly, the core oligosaccharide OSI (10 mg) was oxidised with 0.1 M NaIO_4_ (1 mL) at 21 °C in the dark for 60 min. Then ethylene glycol (10 μL) was added, and the solution was incubated at 21 °C for an additional 60 min. The reaction mixture was then applied directly on the Sephadex G-10 column, equilibrated with 0.05 M pyridine/acetic acid buffer of pH 5.6, and the first eluted fraction was lyophilised. The conjugation was carried out as described previously [20]. The oxidised core oligosaccharide OSI (3.8 mg) dissolved in H_2_O (200 μL) was mixed with an equal volume of BSA solution in H_2_O (2 mg/200 μL). Dimethylformamide was added to a final concentration of 2%, and the mixture was freeze-dried. Dry preparation was heated at 105 °C for 30 min, dissolved in H_2_O (1 mL), and dialyzed against H_2_O. The antigenic properties of the product were analysed by immunoblotting, using polyclonal rabbit serum against *P. shigelloides* PCM 2231.

### 3.7. Immunization Procedures

Rabbits were housed at the animal facility of the Institute of Immunology and Experimental Therapy (Wroclaw, Poland), and all the experiments were carried out according to the procedures approved by the Local Ethical Commission. Rabbits were immunized with the OSI-BSA conjugate, suspended in a complete Freund’s adjuvant, and polyclonal antibodies against the conjugate were obtained by the procedures previously described [31]. 

### 3.8. ELISA

Enzyme-Linked immunosorbent assay (ELISA), using LPS as solid-phase antigen, was performed with a modification [30] of the method described by Voller *et al.* [32]. The detection system consisted of a goat anti-rabbit IgG conjugated with alkaline phosphatase (Bio-Rad, Hercules, CA, USA) as a second antibody and *p*-nitrophenyl phosphate as a substrate.

### 3.9. SDS-PAGE

The LPS was analysed by SDS-PAGE according to the method of Laemmli [33] with modifications described previously [24] and LPS bands were visualised by the silver staining method [21].

### 3.10. Immunoblotting

Immunoblotting was performed on the SDS-PAGE-separated LPS fractions as previously described [34]. A goat anti-rabbit IgG conjugated with alkaline phosphatase (Bio-Rad) was used as the secondary antibody, and 5-bromo-4-chloro-3-indolyl phosphate/nitroblue tetrazolium was applied as a detection system.

## 4. Conclusions

Here we presented the structure of a complete core oligosaccharide composed of an undecasaccharide, which represents the second core type for serotype O17 of *P. shigelloides*. The core OS of *P. shigelloides* PCM 2231 is heterogeneous. The heterogeneity corresponded to the absence of terminal β-D-Glc*p* residue. Three minor glycoforms represent the complete core OS devoid of β-D-Glc*p*NAc residue, both terminal β-D-Glc*p* and hexosamine residues, and both terminal β-D-Glc*p* and β-D-Glc*p*NAc residues.

Serological screening of different strains of *P. shigelloides* indicates that identical or similar epitopes to *P.* *shigelloides* PCM 2231 might also be present in the core region of 28 out of 55 strains (51%). This observation suggests that the core structure in LPS of strain PCM 2231 (serotype O17) could be the most common type in *P. shigelloides*.

Previously published serological studies did not indicate the presence of a common lipopolysaccharide core type of *P. shigelloides*. Similar studies with the use of the antibodies against core OS conjugate were reported for strain *P. shigelloides* 113/92 (serotype O54) indicating serological cross-reactivity was limited to only three strains [8]. This is therefore the first report of the most common core OS structure within *P. shigelloides* LPS. 

## References

[1] Garrity G.M., Bell J.A., Lilburn T.G. Bergey’s Taxonomic Outline. http://dx.doi.org/10.1007/bergeysoutline200310.

[2] Stock I. (2004). *Plesiomonas shigelloides*: An emerging pathogen with unusual properties. Rev. Med. Microbiol..

[3] Linnerborg M., Widmalm G., Weintraub A., Albert M.J. (1995). Structural elucidation of the *O*-antigen lipopolysaccharide from two strains of *Plesiomonas shigelloides* that share a type-specific antigen with *Shigella flexneri* 6, and the common group 1 antigen with *Shigella flexneri* spp. and *Shigella dysenteriae* 1. Eur. J. Biochem..

[4] Czaja J., Jachymek W., Niedziela T., Lugowski C., Aldova E., Kenne L. (2000). Structural studies of the *O*-specific polysaccharide from *Plesiomonas shigelloides* strain CNCTC 113/92. Eur. J. Biochem..

[5] Lukasiewicz J., Dzieciatkowska M., Niedziela T., Jachymek W., Augustyniuk A., Lugowski C., Kenne L.  (2006). Complete lipopolysaccharide of *Plesiomonas shigelloides* O74:H5 (strain CNCTC 144/92) 2. Lipid A, its structural variability, the linkage to the core oligosaccharide, and the biological activity of lipopolysaccharide. Biochemistry.

[6] Lukasiewicz J., Niedziela T., Jachymek W., Kenne L., Lugowski C. (2006). Structure of the lipid A-inner core region and biological activity of *Plesiomonas shigelloides* O54 (strain CNCTC 113/92) lipopolysaccharide. Glycobiology.

[7] Niedziela T., Dag S., Lukasiewicz J., Dzieciatkowska M., Jachymek W., Lugowski C., Kenne L. (2006). Complete lipopolysaccharide of *Plesiomonas shigelloides* O74:H5 (strain CNCTC 144/92). 1. Structural analysis of the highly hydrophobic lipopolysaccharide, including the *O*-antigen, its biological repeating unit, the core oligosaccharide, and the linkage between them. Biochemistry.

[8] Niedziela T., Lukasiewicz J., Jachymek W., Dzieciatkowska M., Lugowski C., Kenne L. (2002). Core oligosaccharides of *Plesiomonas shigelloides* O54:H2 (strain CNCTC 113/92)—Structural and serological analysis of the lipopolysaccharide core region, the *O*-antigen biological repeating unit, and the linkage between them. J. Biol. Chem..

[9] Pieretti G., Corsaro M.M., Lanzetta R., Parrilli M., Canals R., Merino S., Tomás J.M. (2008). Structural studies of the *O*-chain polysaccharide from *Plesiomonas shigelloides* strain 302–73 (serotype O1). Eur. J. Org. Chem..

[10] Pieretti G., Corsaro M.M., Lanzetta R., Parrilli M., Vilches S., Merino S., Tomás J.M. (2009). Structure of the core region from the lippolysaccharide of *Plesiomonas shigelloides* strain 302–73 (serotype O1). Eur. J. Org. Chem..

[11] Pieretti G., Carillo S., Lindner B., Lanzetta R., Parrilli M., Jimenez N., Regue M., Tomas J.M., Corsaro M.M. (2010). The complete structure of the core of the LPS from *Plesiomonas shigelloides* 302–73 and the identification of its *O*-antigen biological repeating unit. Carbohydr. Res..

[12] Maciejewska A., Lukasiewicz J., Niedziela T., Szewczuk Z., Lugowski C. (2009). Structural analysis of the *O*-specific polysaccharide isolated from *Plesiomonas shigelloides* O51 lipopolysaccharide. Carbohydr. Res..

[13] Sawen E., Ostervall J., Landersjo C., Edblad M., Weintraub A., Ansaruzzaman M., Widmalm G. (2012). Structural studies of the *O*-antigenic polysaccharide from *Plesiomonas shigelloides* strain AM36565. Carbohydr. Res..

[14] Kubler-Kielb J., Mocca C., Vinogradov E. (2008). The elucidation of the structure of the core part of the LPS from *Plesiomonas shigelloides* serotype O17 expressing *O*-polysaccharide chain identical to the *Shigella sonnei O*-chain. Carbohydr. Res..

[15] Batta G., Liptak A., Schneerson R., Pozsgay V. (1997). Conformational stabilization of the altruronic acid residue in the *O*-specific polysaccharide of *Shigella sonnei*/*Plesiomonas shigelloides*. Carbohydr. Res..

[16] Kenne L., Lindberg B., Petersson C., Katzenellenbogen E., Romanowska E. (1980). Structural studies of the *O*-specific side chains of the *Shigella sonnei* phase I lipopolysaccharide. Carbohydr. Res..

[17] Aldova E. (1994). Serovars of Plesiomonas shigelloides. Zentralbl. Bakteriol..

[18] Aldova E. (1995). The importance of serotyping *Plesiomonas shigelloides*. Epidemiol. Mikrobiol. Immunol..

[19] Gorin P.A.J., Mazurek M. (1975). Further studies on the assignment of signals in ^13^C magnetic resonance spectra of aldoses and derived methyl glycosides. Can. J. Chem..

[20] Boratynski J., Roy R. (1998). High temperature conjugation of proteins with carbohydrates. Glycoconj. J..

[21] Tsai C.M., Frasch C.E. (1982). A sensitive silver stain for detecting lipopolysaccharides in polyacrylamide gels. Anal. Biochem..

[22] Petersson C., Niedziela T., Jachymek W., Kenne L., Zarzecki P., Lugowski C. (1997). Structural studies of the *O*-specific polysaccharide of *Hafnia alvei* strain PCM 1206 lipopolysaccharide containing D-allothreonine. Eur. J. Biochem..

[23] Westphal O., Jann K. (1965). Bacterial lipopolysacharides: Extraction with phenol-water and further applications of the procedure. Methods Carbohydr. Chem..

[24] Niedziela T., Petersson C., Helander A., Jachymek W., Kenne L., Lugowski C. (1996). Structural studies of the *O*-specific polysaccharide of *Hafnia alvei* strain 1209 lipopolysaccharide. Eur. J. Biochem..

[25] Gerwig G.J., Kamerling J.P., Vliegenthart J.F.G. (1978). Determination of the D and L configuration of neutral monosaccharides by high-resolution capillary GLC. Carbohydr. Res..

[26] Gerwig G.J., Kamerling J.P., Vliegenthart J.F.G. (1979). Determination of the absolute configuration of monosaccharides in complex carbohydrates by capillary GLC. Carbohydr. Res..

[27] Taylor R.L., Shively J.E., Conrad H.E. (1976). Stoichiometric reduction of uronic acid carboxyl groups in polysaccharides. Methods Carbohydr. Chem..

[28] Hakomori S. (1964). A rapid permethylation of glycolipid and polysaccharide catalyzed by methylsulphinyl carbanion in dimethyl sulphoxide. J. Biochem..

[29] Goddard T.D., Kneller D.G. (2001). Sparky.

[30] Jennings H.J., Lugowski C. (1981). Immunochemistry of groups A, B, and C meningococcal polysaccharide-tetanus toxoid conjugates. J. Immunol..

[31] Lugowski C., Romanowska E. (1978). Enterobacterial common antigen: Isolation from *Shigella sonnei*, purification and immunochemical characterization. Eur. J. Biochem..

[32] Voller A., Draper C., Bidwell D.E., Bartlett A. (1975). Microplate enzyme-linked immunosorbent assay for chagas’ disease. Lancet.

[33] Laemmli U.K. (1970). Cleavage of structural proteins during the assembly of the head of bacteriophage T4. Nature.

[34] Lugowski C., Jachymek W., Niedziela T., Rowinski S. (1996). Serological characterisation of anti-endotoxin sera directed against the conjugates of oligosaccharide core of *Escherichia coli* type R1, R2, R3, J5 and *Salmonella Ra* with tetanus toxoid. FEMS Immunol. Med. Microbiol..

